# A Fault Diagnosis Method for Rolling Bearings Based on Enhanced Sparrow Search Algorithm-Optimized VMD and CNN-BiLSTM

**DOI:** 10.3390/s26103239

**Published:** 2026-05-20

**Authors:** Fuqiuxuan Liu, Xiaofeng Yue

**Affiliations:** School of Mechanical and Electrical Engineering, Changchun University of Technology, Changchun 130012, China

**Keywords:** variational mode decomposition, sparrow search algorithm, convolutional neural network, bidirectional long short-term memory

## Abstract

This paper proposes a novel rolling bearing fault diagnosis method to address the difficulty of accurate feature extraction from nonlinear and non-stationary vibration signals. First, a Levy–Cauchy Optimized Sparrow Search Algorithm (LOCSSA) is developed to optimize the two core parameters (decomposition level and penalty factor) of Variational Mode Decomposition (VMD), and the optimized VMD is used to decompose raw vibration signals to obtain optimal intrinsic mode functions (IMFs). Second, the extracted IMF features are fed into a convolutional neural network (CNN) for local pattern extraction, followed by a bidirectional long short-term memory (BiLSTM) network to model temporal dependencies, with the final fault classification completed via a fully connected layer. Comparative experiments and ablation studies with five benchmark models are conducted to verify the effectiveness of the proposed framework. The results show that the proposed method achieves 96.33% accuracy, 96.67% recall, and 96.54% F1-score, outperforming all benchmark models. Ablation analysis confirms that both LOCSSA-optimized VMD and BiLSTM contribute significantly to performance improvement (*p* < 0.05), validating the rationality of the proposed method.

## 1. Introduction

Rolling bearings serve as critical components in rotating machinery systems, with their widespread deployment across modern industrial applications. Owing to their complex operating environments, variable loading conditions, and sustained high-frequency operation, rolling bearings are particularly susceptible to performance degradation and failure modes during extended service periods. With the continuous accumulation of vibration and shock, severe faults may result in unplanned machine downtime and substantial economic losses. Therefore, research on bearing fault diagnosis holds significant engineering-related importance for ensuring operational reliability and preventing catastrophic failures in industrial systems [1].

In recent years, with the rapid development of intelligent technologies, sensorless condition monitoring has become a cutting-edge research direction in the field of rotating machinery fault diagnosis. This type of method does not require direct contact with components, effectively solves the problem of limited deployment of contact sensors in working conditions such as narrow spaces and high-speed rotation, and can realize long-distance monitoring through acoustic signals, motor torque signals, etc. Meanwhile, in the research on condition monitoring of wind turbine gearboxes and bearings, the systematic comparison and optimization of diagnostic indicators have become core research hotspots, and machine learning technology has been used to study the adaptability and sensitivity of different time-domain and frequency-domain indicators under variable working conditions and strong noise environments. It is worth noting that although sensorless condition monitoring has made significant progress in recent years, this technology combined with the enhanced Weighted K-Nearest Neighbors (WKNN) algorithm can improve the sensitivity to early degradation [2,3]. Although the above emerging methods have shown new research ideas, vibration signal analysis, by virtue of its high sensitivity to changes in mechanical condition monitoring and fault impact characteristics, is still the most mature and widely used core technical means in the field of rolling bearing fault diagnosis at present.

In the condition monitoring of rolling bearings, the effective extraction and analysis of vibration signals are critical for accurate fault diagnosis. However, owing to periodic impacts, time-varying operating conditions, environmental noise, and external disturbances, these signals exhibit strong non-stationary and nonlinear characteristics [4]. In the field of signal feature extraction, several widely used methods include Empirical Mode Decomposition (EMD) [5], Ensemble Empirical Mode Decomposition (EEMD) [6], Complementary Ensemble Empirical Mode Decomposition (CEEMD) [7], and Local Mean Decomposition (LMD) [8]. However, these methods are inherently constrained by their respective limitations. For instance, when processing vibration signals, EMD often suffers from mode mixing, boundary effects, and high sensitivity to noise [9]. EEMD was developed to mitigate mode mixing by introducing Gaussian white noise; however, this approach introduces noise-dependent decomposition, substantial reconstruction errors, and high computational costs [10]. Although CEEMD mitigates mode mixing to a certain extent, it fails to eliminate it completely. Moreover, CEEMD requires multiple iterative computations, resulting in significantly prolonged processing time. Additionally, the decomposition results may demonstrate increased uncertainty owing to amplified noise interference [11]. In the LMD method, the decomposition outcomes are highly sensitive to parameter selection, leading to potential inconsistencies in the results [12].

To overcome the limitations of the aforementioned methods, VMD was introduced in 2014 [13]. Owing to its capability to decompose non-stationary and nonlinear signals while precisely separating modal functions of distinct temporal scales, VMD has been extensively applied across diverse fields such as biology [14], signal processing [15], materials science [16], and energy systems [17]. However, the performance of VMD critically depends on the appropriate selection of its parameters, including the number of decomposition modes and the penalty factor. An inappropriate number of modes may result in either over-decomposition, where extraneous components are generated, or under-decomposition, where distinct modes remain inadequately separated. The penalty factor primarily governs the trade-off between the data fidelity and the smoothness of the extracted IMF components by imposing bandwidth constraints. An excessively large penalty factor tends to induce over-smoothing of the IMFs, which may lead to the loss of critical transient features and subtle fault signatures in the signal. Conversely, an insufficient penalty factor may fail to adequately suppress noise, amplifying interference components and reducing the decomposition accuracy due to excessive mode overlap [18,19].

To address the limitation of fixed parameter matching of VMD in engineering fault diagnoses, scholars have carried out extensive research on adaptive parameter optimization methods, which can be mainly divided into two core technical routes.

The first route is statistical feature indicator-driven adaptive parameter optimization, which uses fault-sensitive statistical indicators as the objective function to directly optimize the key parameters of VMD. Lei et al. [20] developed a global optimization algorithm for VMD that utilizes kurtosis indicators to adaptively determine the optimal decomposition level and penalty factor. However, under strong background noise, its decomposition results are prone to mode mixing or spurious components, which reduces the accuracy of fault feature extraction. Li et al. [21] proposed a VMD-based Mode Extraction (VME) method, which optimizes the penalty factor with kurtosis as the objective function to enhance the separation of fault-related modes, and achieved good performance in multi-fault diagnosis. Li et al. [22] employed maximum envelope kurtosis as the optimization indicator for VMD parameters, and selected effective IMFs based on frequency band entropy, which was validated by engineering cases. Wei et al. [23] introduced a fault diagnosis framework integrating Composite Scale Variational Dispersion Entropy (CSvDE) with Self-Optimizing VMD (SoVMD), realizing adaptive signal decomposition and enhanced feature extraction.

The second route is swarm intelligence algorithm-based global parameter optimization, which takes fault-sensitive indicators as the fitness function and uses swarm intelligence algorithms to realize the global optimal search of VMD parameters. Guo et al. [24] explored an enhanced Gravitational Search Algorithm (GSA) for VMD parameter optimization, but this method is highly dependent on the initial parameter settings and is prone to premature convergence. Li et al. [25] introduced an enhanced deep learning method incorporating the Beetle Antennae Search (BAS) algorithm, which was verified to be effective in diagnosing various bearing faults. Zhou et al. [26] developed a modified Particle Swarm Optimization (PSO) algorithm to optimize VMD parameters, and combined it with the Cyclic Autocorrelation Function (CAF) to enhance composite fault feature extraction, with effectiveness validated by simulation and measured signal analysis. Zhou et al. [27] proposed a hybrid method combining the Whale Optimization Algorithm (WOA) and Grey Wolf Optimizer (GWO) to optimize key VMD parameters, and adopted Support Vector Machines (SVMs) for fault classification; however, their comparative analysis is relatively limited, lacking comprehensive benchmarking with other state-of-the-art swarm intelligence algorithms.

For the optimization of VMD parameters, existing studies have investigated various algorithms, including PSO [28], GWO [29], and the Genetic Algorithm (GA) [30]. Hang et al. [31] employed the SSA with sample entropy as the objective function to optimize VMD parameters (decomposition level K and penalty factor α); however, their approach neglected SSA’s inherent tendency to converge to local rather than global optima. Li et al. [32] further enhanced feature extraction by integrating GA-optimized VMD with center frequency analysis, enabling the more precise isolation of fault-related frequency components.

Shen et al. [33] developed an AGWO-PSO-VMD-TEFCG-AlexNet method for strong noise bearing fault diagnosis, using envelope entropy as the optimization objective. Shen et al. [34] applied WOA-VMD combined with stacked LSTM to construction noise prediction, and adopted the novel Weighted Quality Evaluation (WQE) index for model evaluation.

Compared to conventional optimization algorithms, the SSA is a swarm intelligence-based method inspired by the collective foraging and anti-predation behaviors of sparrows, characterized by its rapid convergence and strong global optimization capabilities. The algorithm’s principles are intuitive and computationally efficient, facilitating straightforward implementation. SSA has exhibited outstanding search performance and rapid convergence rates in addressing optimization problems, allowing it to swiftly identify near-optimal solutions [35]. Furthermore, its inherently parallel structure enables the efficient utilization of multi-core processors or distributed computing resources, significantly accelerating the solution process. Despite these advantages, SSA is not without limitations. However, SSA tends to converge to local optima, and its theoretical underpinnings remain relatively underdeveloped, with limited comprehensive studies validating its efficacy in real-world engineering applications.

Deep learning has demonstrated substantial advancements in the field of rolling bearing fault diagnosis, offering enhanced capabilities in feature extraction, pattern recognition, and predictive accuracy. Deep learning models, particularly architectures such as CNN [36] and recurrent neural networks (RNNs) [37], have been extensively utilized for analyzing bearing fault data, leveraging their ability to autonomously extract discriminative features from raw vibration signals and operational data. Wang et al. [38] introduced a fault diagnosis method for rolling bearings integrating VMD with deep learning techniques. In contrast to conventional diagnostic approaches, their method demonstrated substantial enhancements in recognition accuracy, robustness to noise, and generalization capability across diverse operational conditions. Li et al. [39] developed an integrated approach combining VMD with transfer learning, effectively addressing the limitations of conventional methods that rely heavily on large labeled datasets. This method significantly enhances fault diagnosis accuracy and generalization capability by leveraging knowledge transfer across different operational conditions. Lu et al. [40] developed a CNN framework that integrates image recognition techniques and visual feature extraction capabilities into bearing fault diagnosis, enabling the automated and high-precision identification of fault patterns. Appana et al. [41] enhanced bearing fault classification accuracy by extracting discriminative features from acoustic emission (AE) signals via envelope spectrum analysis and CNN. Building on this line of research that combines deep learning with vibration/acoustic signal feature engineering, the present study proposes a novel hybrid framework integrating CNN with BiLSTM, which enables comprehensive spatiotemporal feature learning from bearing fault data, thus achieving more robust and accurate fault diagnosis.

The literature review reveals significant research potential in the optimization of VMD parameters, particularly in addressing the limitations of existing methods to achieve more adaptive and accurate fault feature extraction. This paper proposes a novel fault diagnosis method that integrates VMD optimized by LOCSSA with a hybrid CNN-BiLSTM architecture to improve the accuracy and robustness of rolling bearing fault detection. The vibration signals of rolling bearings are first decomposed using VMD. To adaptively determine the optimal VMD parameters—the number of modes (K) and the penalty factor (α)—LOCSSA is employed.

The rolling bearing vibration signals are decomposed into a set of IMFs using VMD optimized by the LOCSSA. To evaluate the quality of the decomposition, Permutation Entropy (PE) and Mutual Information Entropy (MIE) are extracted and fused as a multi-entropy feature set, serving as a joint objective function for assessing the informativeness and regularity of the IMFs.

For the VMD results optimized by LOCSSA, spurious components are eliminated by calculating their Pearson correlation coefficients with the original signal, and the remaining informative IMFs are reconstructed to form a denoised and enhanced signal representation. Nine time-domain statistical features—namely mean, variance, kurtosis, skewness, root mean square (RMS), peak factor, impulse factor, waveform factor, and clearance factor—are extracted from the screened and reconstructed informative IMFs to form high-dimensional feature vectors for downstream fault diagnosis. A hybrid CNN-BiLSTM model is adopted as the fault diagnosis framework. The extracted high-dimensional feature vectors are fed into this network to automatically learn discriminative spatiotemporal patterns for accurate fault classification.

The efficacy of the proposed fault diagnosis method is validated through comprehensive experiments utilizing both simulated signals and the publicly available Case Western Reserve University (CWRU) rolling bearing dataset. The results confirm its robustness and practical utility in real-world engineering scenarios. This study selects rolling bearings as the typical research subject to validate the effectiveness of the proposed LOCSSA-VMD-CNN-BiLSTM fault diagnosis framework. Notably, the established framework is not limited to the inherent fault characteristics of rolling bearings. Constructed by integrating adaptive signal decomposition with deep learning, the presented model exhibits excellent generalization capability. With slight parameter adjustments, it can be readily extended to fault diagnosis applications for other vibration-dependent mechanical equipment, including gear transmission systems.

The remainder of this paper is organized as follows: Section 2 presents the theoretical foundations of VMD, SSA, CNN, and BiLSTM. Section 3 elaborates on the proposed LOCSSA-VMD-CNN-BiLSTM framework, including its architecture and implementation details. Section 4 validates the effectiveness of the method through experiments based on the publicly recognized CWRU bearing dataset. Finally, Section 5 summarizes the study and suggests potential future research directions.

## 2. Related Theoretical Foundation

### 2.1. VMD Algorithm

The VMD method decomposes the original signal f(x) into K IMFs $u_{k}(t)$, each characterized by a central frequency $\omega_{k}(t)$. This method redefines IMFs as band-limited amplitude-modulated and frequency-modulated (AM-FM) signals, ensuring the adaptive separation of complex vibration components.

(1) $$u_{k}(t)=A_{k}(t)\cos(\varphi_{k}(t)),\;$$

where $A_{k}(t)\geq 0$ represents the instantaneous amplitude of $u_{k}(t)$; $\omega_{k}(t)$ denotes the instantaneous frequency of $u_{k}(t)$; and $\phi_{k}(t)$ is a non-decreasing phase function, where both $A_{k}(t)$ and $\omega_{k}(t)$ vary slowly with respect to $\phi_{k}(t)$.

The estimation of bandwidth for each IMF is formulated as a constrained variational model, optimized through the following steps:

1. For each mode function $u_{k}(t)$, the Hilbert transform is applied to obtain its analytic signal, thereby facilitating the derivation of its unilateral frequency spectrum.
2. Frequency Shifting: The analytic signal is frequency-shifted by mixing it with an exponential term $e^{-j\omega_{k}t}$, which translates its spectrum to a baseband centered around zero frequency.
3. Bandwidth Calculation: The bandwidth of each mode is quantified by computing the squared *L*^2^-norm of the time gradient of the demodulated analytic signal, representing the mode’s frequency-domain compactness.

The constrained variational model for estimating the bandwidth of all modes is formulated as follows:

(2) $$\left.\begin{array}{c} \underset{\left\{u_{k}\},\{\omega_{k}\right\}}{\min}\left\{\sum_{k}{\left\|\partial_{t}\left[\left(\delta (t)+\frac{j}{\pi t}\right)*u_{k}(t)\right]e^{-j\omega_{k}t}\right\|}_{2}^{2}\right\}\\ s.\;t.\;\sum_{k=1}^{K}u_{k}=f(t) \end{array}\right\},\;$$

#### Parameter Sensitivity Analysis

Applying VMD to bearing vibration signals yields a set of IMFs, each representing an adaptively extracted oscillatory mode from the raw signal. The decomposition performance of VMD is highly sensitive to two critical parameters: the mode number K and the penalty factor α, as inappropriate parameter selection tends to cause spurious components, mode mixing, or incomplete decomposition.

**Figure 1:**
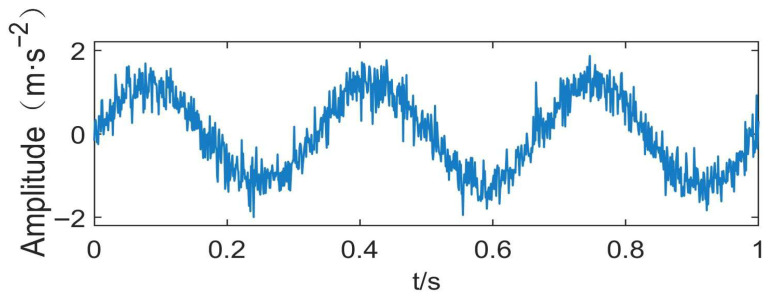
Time-domain waveform of the simulated vibration signal.

To investigate the impact of parameter selection on decomposition performance, a simulated signal is constructed to mimic the typical vibration characteristics of localized point defect faults in rolling bearings, which are the most common and representative fault types in industrial rotating machinery, including inner race faults, outer race faults, and rolling element faults. These faults share the common characteristic of generating periodic transient impulse signals, which is the core feature that VMD aims to extract and separate. Its time-domain waveform is shown in Figure 1.

The analysis of the autocorrelation coefficients and kurtosis of the resulting IMFs verifies that the rational selection of K and α is essential to guarantee the accuracy of VMD. The kurtosis and correlation coefficient values under different K and α values are summarized in Table 1.

The mathematical expression of the simulated signal is given as follows:

(3) $$x(t)=\sum_{k=1}^{3}A_{k}\cos(2\pi f_{k}t),\;$$

where $f_{1}=2\;\mathrm{Hz},\;A_{1}=1$, $f_{2}=4\;\mathrm{Hz},\;\;A_{2}=0.25$, $\varepsilon (t)∼Ν(0,\;\delta^{2})$. The frequency ratio is set to 1:12:144 to evaluate the algorithm’s resolution for broadband signal decomposition. Gaussian white noise is added to construct the observed noisy signal:

(4) f(t)=x(t)+ε(t), 

where $\varepsilon (t)∼Ν(0,\;\delta^{2})$, δ=0.1, corresponding the signal-to-noise ratio, as follows:

(5) $$SNR=10\log_{10}\left(\frac{P_{signal}}{P_{noise}}\right)=10\;\mathrm{dB}.$$

**Figure 2:**
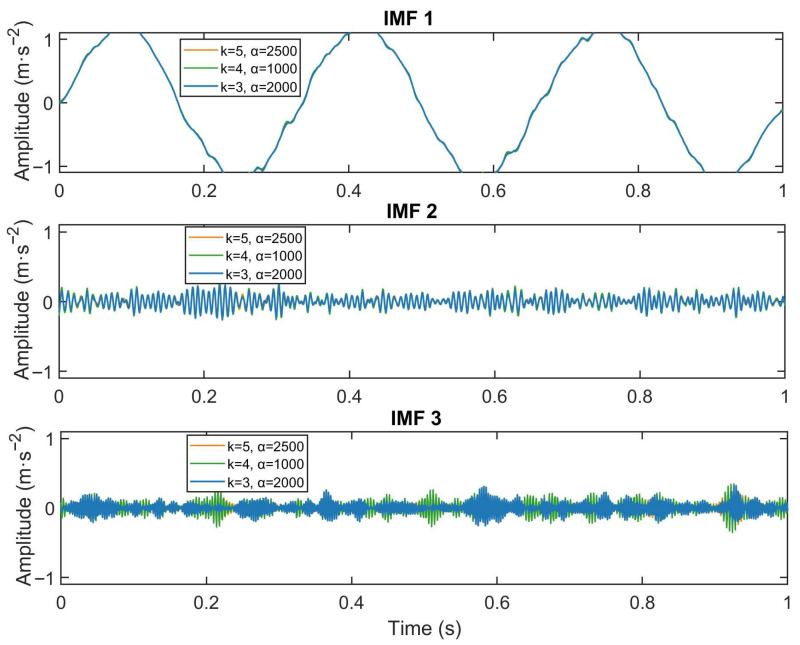
Comparison of the first three IMFs from VMD with (blue) K=3, α=2000; (green) K=4, α=1000; and (orange) K=5, α=2500. The vertical axis is standardized to [−1, 1].

Select three sets of values for (K=5, α=2500), (K=3, α=2000), and (K=4, α=1000). VMD was performed on the signal, followed by the calculation of the correlation coefficients and kurtosis values of the resulting IMFs to assess their relevance to the original signal and impulsivity. The comparison results of the first three IMFs obtained under different parameter combinations are shown in Figure 2.

The decomposition profiles, supported by high kurtosis values and dominant autocorrelation coefficients, collectively demonstrate that the parameter combination K=3, α=2000 achieves precise modal separation, effectively isolating both the principal oscillatory component and transient impacts. Specifically, IMF1 exhibits low kurtosis and high autocorrelation, indicating a deterministic component. In contrast, IMF2 shows high kurtosis with low autocorrelation, characteristic of transient impulses, whereas IMF3 retains residual noise, suggesting incomplete denoising.

IMFs 3–5 exhibit intermediate-to-high kurtosis values, suggesting the coexistence of meaningful signal components and residual noise in these modes. This result indicates that such parameter settings lack sufficient discriminative capacity to effectively separate noise from fault-related components. Conversely, with parameter set K=5, α=2500, IMFs 3–5 exhibit moderate kurtosis alongside low autocorrelation values, indicating the presence of spurious components or residual noise—a characteristic signature of over-decomposition.

Notably, an insufficient number of modes K leads to under-decomposition, whereas an inadequately small penalty factor (e.g., α=1000) increases sensitivity to noise and compromises mode compactness. Increasing the penalty factor to α=2500 improves noise suppression but may also lead to signal over-smoothing and the loss of transient features. These results emphasize the necessity of jointly optimizing both the mode number K and penalty factor α to achieve an effective compromise between component fidelity and noise robustness.

The simulated signal analysis confirms that improper parameter selection in VMD significantly compromises decomposition quality, manifesting as either residual noise in the extracted modes or the generation of artificial components lacking physical meaning. Consequently, achieving an optimal balance between the mode number K and penalty factor α is essential for ensuring robust VMD performance in rolling bearing fault diagnosis.

### 2.2. LOCSSA

The SSA is a swarm intelligence-based optimizer that mimics the collective foraging and anti-predation behaviors of sparrows. Its search process is driven by the coordinated roles of producers, scroungers, and scouts, which collaboratively enable efficient global exploration and local refinement.

Position Update for Producers:

(6) $$X_{i,j}^{t+1}=\left\{\begin{array}{ll} X_{i,j}^{t}\cdot \exp(\frac{-i}{\alpha \cdot {iter}_{\max}}), & R_{2}<ST\\ X_{i,j}^{t}+Q\cdot L, & R_{2}\geq ST, \end{array}\right.$$

where *t* denotes the current iteration, $X_{i,j}^{t}$ represents the position of the *i*-th sparrow in the *j*-th dimension at iteration t, $iter_{\max}$ is the maximum number of iterations, $R_{2}\in [0,\;1]$ is a random threshold parameter, and ST∈[0.5, 1.0] is the safety threshold.

When the condition $R_{2}<ST$ satisfied, the absence of immediate predator threats enables producers to conduct extensive global exploration for promising regions. Otherwise, detected dangers trigger alarm signals, prompting scroungers and scouts to execute evasive maneuvers through Lévy-distributed random walks for rapid hazard avoidance.

The position update rule is

(7) $$X_{i,j}^{t+1}=\left\{\begin{array}{cc} Q\cdot \exp(\frac{X_{worst}^{t}-X_{i,j}^{t}}{i^{2}}), & i>\frac{n}{2}\\ X_{p}^{t+1}+\left|X_{i,j}^{t}-X_{p}^{t+1}\right|\cdot A^{+}\cdot L, & i\leq \frac{n}{2}, \end{array}\right.$$

where $X_{p}^{t+1}$ denotes the global best position discovered by the sparrow producers at the (t+1)−th generation. $X_{worst}^{t}$ represents the global worst position from the previous (t−th) generation. A is a 1×m matrix satisfying the pseudoinverse relation $A^{+}=A^{T}(AA^{T}{)}^{-1}$.

The position update mechanism for vigilant individuals (scouts) is mathematically formulated as

(8) $$X_{i,j}^{t+1}=\left\{\begin{array}{cc} X_{best}^{t}+\beta \cdot \left|X_{i,j}^{t}-X_{best}^{t}\right|, & f_{i}>f_{g}\\ X_{i,j}^{t}+k\cdot \frac{\left|X_{i,j}^{t}-X_{best}^{t}\right|}{f_{i}-f_{w}+\varepsilon }, & f_{i}=f_{g}, \end{array}\right.$$

where X denotes the global best position within the sparrow population at the t−th generation. β and k are control coefficients for adjusting the step size, where k∈[−1, 1], and ε is a constant. If the fitness value of the n−th sparrow $f_{i}$ exceeds the global best fitness $f_{g}$, this indicates that the sparrow is located at the edge of the population and actively moves toward the global best position to refine its solution. If $f_{i}=f_{g}$, the sparrow is in a dangerous position and must escape by relocating away from the global worst position.

The conventional SSA is prone to several limitations, such as sensitivity to parameter settings, suboptimal population diversity, slow convergence rates, and a tendency to converge to local optima, which collectively constrain its optimization performance. To overcome these limitations, this paper introduces an enhanced Sparrow Search Algorithm that strategically integrates Levy flight and Cauchy mutation strategies, forming the LOCSSA.

The proposed LOCSSA is a pragmatically integrated metaheuristic, specifically designed for the VMD parameter optimization task in vibration signal analysis. It synergistically combines four enhancement strategies to achieve a superior balance between exploration and exploitation, thereby improving the quality of feature extraction.

1. Cubic–Sine Chaotic Initialization

The initial population of sparrows is generated via a Cubic–Sine compound chaotic mapping, expressed as

(9) $$x_{k+1}=\sin(\pi (4\theta x_{n}(1-x_{n}^{2}))),$$

where θ denotes the control parameter with θ∈(0, 1), and $x_{n}\in (0,\;1)$ represents the chaotic state at the n−th iteration.

2. Osprey-inspired Global Exploration

The producers incorporate the dynamic hunting mechanism from the Osprey Optimization Algorithm (OOA) [42], updating their positions as follows:

(10) $$X_{i,j}^{t+1}=\left\{\begin{array}{cc} X_{i,j}^{t+1}=X_{i,j}^{t}+r_{i,j}\cdot (SF_{i,j}-I_{i,j}\cdot X_{i,j}^{t}), & R_{2}<ST\\ X_{i,j}^{t}+Q\cdot L, & R_{2}\geq ST. \end{array}\right.$$

3. Hybrid Mutation for Local Refinement

To mitigate premature convergence, followers utilize a Cauchy hybrid mutation strategy that perturbs their positions using the heavy-tailed Cauchy distribution, enhancing the ability to escape local optima.

(11) $$X_{i,j}^{t+1}=X_{i,j}^{t}+X_{i,j}^{t}\cdot Cauchy(0,1).$$

4. Levy-flight-assisted Local Refinement

To further balance global exploration and local exploitation, Levy flight is introduced to realize long-range exploration and fine-grained local search. It broadens the algorithm’s search range and enables the population to escape local stagnation regions, whose position-update rule is expressed as follows:

(12) $$X_{i,j}^{t+1}=\left\{\begin{array}{cc} X_{best}^{t}+\alpha Levy(\beta )\cdot \left|X_{i,j}^{t}-X_{best}^{t}\right|, & f_{i}>f_{g}\\ X_{i,j}^{t}+k\cdot \frac{\left|X_{i,j}^{t}-X_{best}^{t}\right|}{f_{i}-f_{w}+\varepsilon }, & f_{i}=f_{g}. \end{array}\right.$$

**Figure 3:**
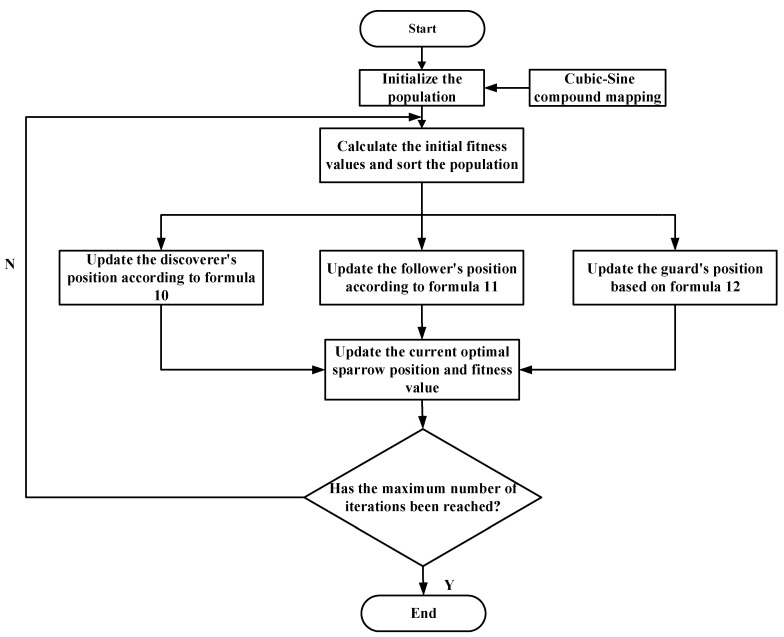
Flowchart of the proposed LOCSSA.

The convergence of LOCSSA is ensured by the orchestrated synergy of its multi-stage search mechanisms, which collectively balance global exploration and local exploitation. The composite chaotic map enhances initial population diversity by leveraging the inherent ergodicity and randomness of chaotic systems, thereby effectively preventing premature convergence to local optima. The optimization framework synergistically integrates three core strategies: the Osprey Optimization Algorithm guides the swarm toward promising regions, Levy flight governs long-range exploration coupled with local refinement, and Cauchy mutation injects perturbations to escape saddle points. Figure 3 depicts the complete optimization flowchart of the proposed LOCSSA.

### 2.3. Parameter Optimization of VMD Using LOCSSA

As demonstrated by the preceding analysis, the decomposition performance of VMD is critically determined by two core parameters: the number of modes K and the penalty factor α. Therefore, the systematic optimization of these two parameters is essential to ensure accurate decomposition of rolling bearing vibration signals and subsequent reliable fault diagnosis. In this paper, the LOCSSA is adopted to simultaneously optimize K and α, with an adaptive selection mechanism to identify the optimal parameter combination.

Permutation Entropy (PE) and Mutual Information (MI) are selected as the joint optimization objectives, owing to their complementary advantages in evaluating signal characteristics. PE quantifies the dynamic complexity of signals and effectively detects non-stationary transient components, while MI measures the statistical dependence between signal components to suppress mode mixing, capturing nonlinear correlations that PE may overlook [43]. By fusing PE and MI, VMD can be optimized simultaneously in terms of dynamic complexity and statistical independence, a strategy widely validated in existing signal separation studies [44,45]. The specific weighting scheme is formulated as follows:

(13) Fitness=α×Norm(PE)+β×Norm(MI), 

where the weight coefficients a=0.6 and b=0.4 are determined through grid search.

### 2.4. Fault Diagnosis Model Based on CNN-BiLSTM

CNN is a classic deep learning model with strong local feature extraction capability. It effectively captures transient impact features in vibration signals via hierarchical convolution and pooling operations, making it ideal for mechanical fault signals with local anomalies.

BiLSTM is an improved recurrent neural network that captures both forward and backward temporal context. It addresses the long-term dependency and gradient vanishing issues of traditional RNNs, and excels at processing sequential vibration signals.

**Figure 4:**
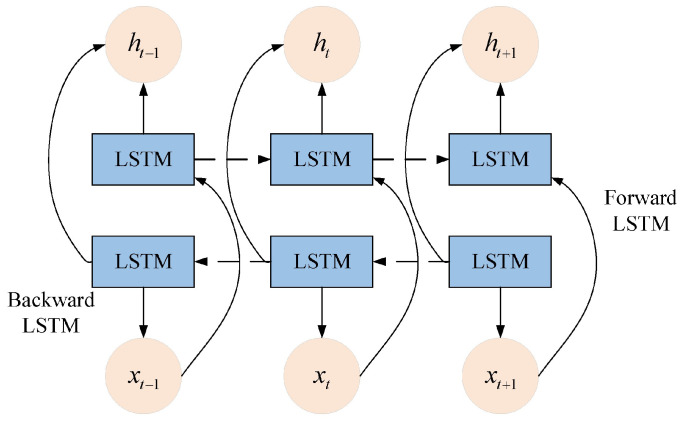
Structure diagram of BiLSTM. Forward LSTM and Backward LSTM denote forward and backward long short-term memory layers, respectively; arrows represent the transmission direction of sequential information, $x_{t}$ is the input feature at time step t, and $h_{t}$ is the corresponding hidden-state output.

In a BiLSTM network, the output at each time step t is formed by concatenating the hidden states from the forward LSTM ($h_{t}^{f}$) and the backward LSTM ($h_{t}^{b}$). The architecture of the BiLSTM is illustrated in Figure 4. The mathematical formulation of this process is as follows:

(14) $$h_{t}=\left[h_{t}^{f},\;h_{t}^{b}\right].$$

**Figure 5:**
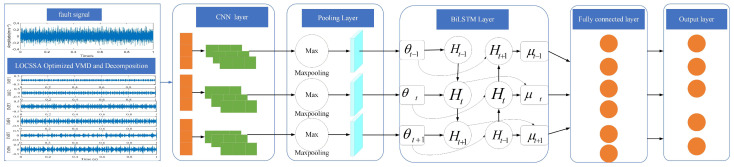
Rolling bearing fault model based on CNN-BiLSTM.

Combining the local convolution operation of CNN to effectively extract transient impacts in vibration signals and the characteristic of BiLSTM to enhance the model’s utilization of time series, this paper proposes a CNN-BiLSTM fault diagnosis model for the diagnosis of rolling main bearing faults. The model structure is shown in Figure 5.

## 3. A Comprehensive Framework: LOCSSA-VMD-CNN-BiLSTM for Fault Diagnosis

**Figure 6:**
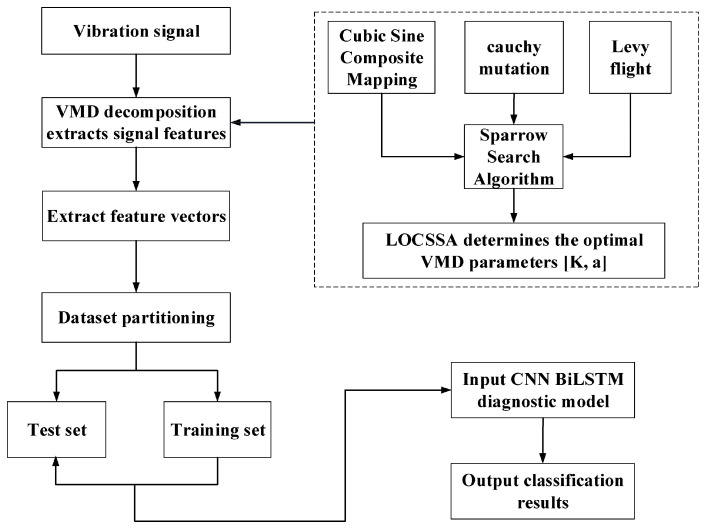
Flowchart of the proposed LOCSSA-VMD-CNN-BiLSTM fault diagnosis framework.

The overall workflow of the proposed LOCSSA-VMD-CNN-BiLSTM fault diagnosis framework is schematically depicted in Figure 6. The framework consists of five core stages, which are elaborated as follows:

(1) Signal Acquisition

Collect vibration acceleration signals from rolling bearings under different health states and operating conditions using piezoelectric accelerometers.

(2) LOCSSA-based VMD Parameter Optimization

To achieve optimal VMD performance, the decomposition level K and penalty factor α are iteratively optimized using the proposed LOCSSA. The algorithm is initialized with a predefined population size, maximum iterations and parameter search ranges. The sparrow population is initialized via Cubic–Sine chaotic mapping to improve diversity, and the joint Permutation Entropy–Mutual Information metric is used as the fitness function to evaluate VMD quality. Iteratively update the positions of producers, followers and scouts to obtain the optimal VMD parameter combination.

(3) Signal Decomposition and Feature Extraction

Decompose the raw vibration signals into denoised, physically meaningful intrinsic mode functions (IMFs) using the LOCSSA-optimized VMD. Extract multi-domain features from the IMFs to construct feature vectors, and divide the dataset into training and test subsets.

(4) CNN-BiLSTM Feature Learning

Feed the IMF feature matrix into the CNN to extract local spatial patterns and perform hierarchical feature fusion. The refined features are then input into the BiLSTM network to capture long-range temporal dependencies and contextual information in the sequence.

(5) Fault Classification and Identification

Apply the high-level features from BiLSTM to the output space via a fully connected layer, and use the Softmax classifier to predict fault categories. The trained model is tested on the test set to realize automated rolling bearing fault diagnosis.

## 4. Experimental Validation and Analysis

### 4.1. Dataset Construction and Description

**Figure 7:**
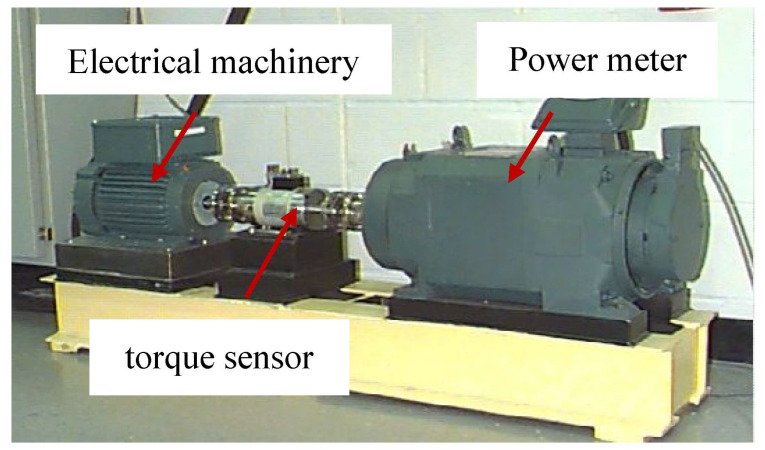
Experimental setup of the CWRU rolling bearing test rig.

To evaluate the performance of the proposed LOCSSA-VMD-CNN-BiLSTM fault diagnosis framework, experiments were performed using the publicly available rolling bearing fault dataset from CWRU [46]. The experimental test rig, illustrated in Figure 7, comprises a 2 hp motor, a torque transducer/encoder, and a power analyzer, which collectively simulate typical industrial operating conditions.

The experiments utilized a 6205-2RS JEM SKF deep groove ball bearing, with vibration signals acquired at a sampling frequency of 12 kHz. Data from the drive end were extracted for analysis. The experiments utilized a 6205-2RS JEM SKF deep groove ball bearing, with vibration signals acquired at a sampling frequency of 12 kHz. Data from the drive end were extracted for subsequent analysis. All tests were conducted under four levels of radial loads (0 hp, 1 hp, 2 hp, and 3 hp), corresponding to approximate motor speeds of 1797 rpm, 1772 rpm, 1750 rpm, and 1730 rpm respectively. Detailed technical specifications of the bearing are summarized in Table 2.

Vibration signals were collected under four distinct health conditions: normal state, inner race fault, rolling element fault, and outer race fault. A total of ten state-specific datasets were acquired, with each condition comprising 120 samples.

**Figure 8:**
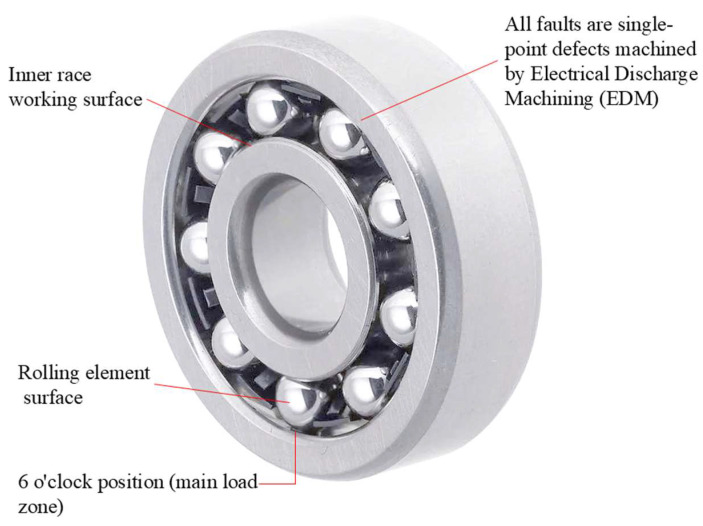
Schematic diagram of artificial fault machining positions for rolling bearings.

All faults were artificially introduced using Electrical Discharge Machining (EDM) in the load-carrying zones of bearing components, which are the regions most susceptible to fatigue damage during operation. Inner race and rolling element faults were machined directly on their respective working surfaces, while outer race faults were introduced at the 6 o’clock position (the primary load zone) of the outer ring. A schematic diagram illustrating the fault machining locations is presented in Figure 8.

Three fault diameter levels were used in the experiments: 0.007 inches (0.178 mm), 0.014 inches (0.356 mm), and 0.021 inches (0.533 mm). These fault sizes were selected to simulate the early, middle, and late stages of bearing degradation, which are the standard fault severity levels widely adopted in the field of bearing fault diagnosis. In general, larger fault sizes produce stronger transient impulse signals with more distinct fault characteristics, whereas smaller early-stage faults generate weaker vibration features that are more easily masked by background noise.

Each health state corresponds to single-point independent damage on a single bearing, and no multi-fault coexistence conditions were included in this study. A total of ten test bearings were used in the experiments, with one bearing dedicated to each operating condition. It should be noted that the diagnosis of multi-fault coupling conditions in real industrial scenarios remains a challenging research problem, and the adaptability and performance of the proposed method under such complex operating conditions will be further investigated as the focus of our future work.

Each raw signal segment was divided into samples of 2048 data points. To assess the model’s generalization ability, a stratified 5-fold cross-validation strategy was adopted. The complete dataset was partitioned into five balanced subsets, ensuring that the proportion of samples from each fault category was preserved in every fold. In each iteration, one subset served as the test set while the remaining four were used for training. To ensure reproducibility, a fixed random seed (seed = 42) was applied during the data splitting process. This entire procedure was repeated independently five times, with the final performance metrics reported as the mean and standard deviation across all runs.

**Figure 9:**
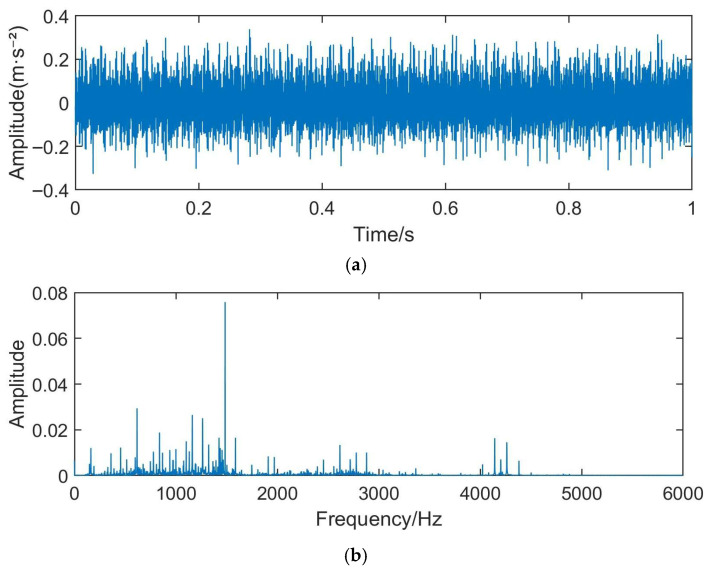
Signal representations of a bearing fault case: (**a**) time-domain waveform; (**b**) frequency-domain spectrum.

The detailed descriptions of the ten health states of rolling bearings are provided in Table 3. Figure 8 shows the schematic diagram of artificial fault machining positions for rolling bearings, which corresponds one-to-one with the fault types listed in Table 3. Figure 9 presents the time-domain waveform and corresponding frequency spectrum of a vibration signal under the inner race fault condition. As can be observed, conventional Fourier transform-based spectral analysis cannot clearly reveal the characteristic fault frequencies, highlighting the limitation of traditional methods in processing such non-stationary signals.

### 4.2. LOCSSA-VMD Parameter Optimization and Feature Extraction

This study employs the LOCSSA-optimized VMD method for fault feature extraction from rolling bearing vibration signals. The underlying principles and algorithmic enhancements of LOCSSA and VMD have been detailed in previous sections. The procedure consists of two main stages:

1. Parameter Optimization: The composite measure of Permutation Entropy and mutual information is used as the fitness function to optimize the VMD parameters, yielding the optimal decomposition level (K) and penalty factor (α).
2. Optimal Mode Extraction: The optimized parameters are then applied to perform VMD, from which the IMFs corresponding to the extremum of the composite entropy objective are selected as the most informative fault-related components.

To verify the optimization effectiveness of the proposed LOCSSA on VMD parameters, the above-mentioned drive end bearing fault data file 105.mat with a sampling frequency of 12 kHz is selected. The fault type is inner race fault (fault diameter 0.007 inches), the motor speed is 1797 rpm, and the load is 0 HP. The classic VMD adopts the default parameters widely used in existing studies (number of modes *K* = 4; penalty factor α = 2000), while the LOCSSA-optimized VMD adaptively obtains the optimal parameter combination for this signal through the optimization process described in Section 2.2.

**Figure 10:**
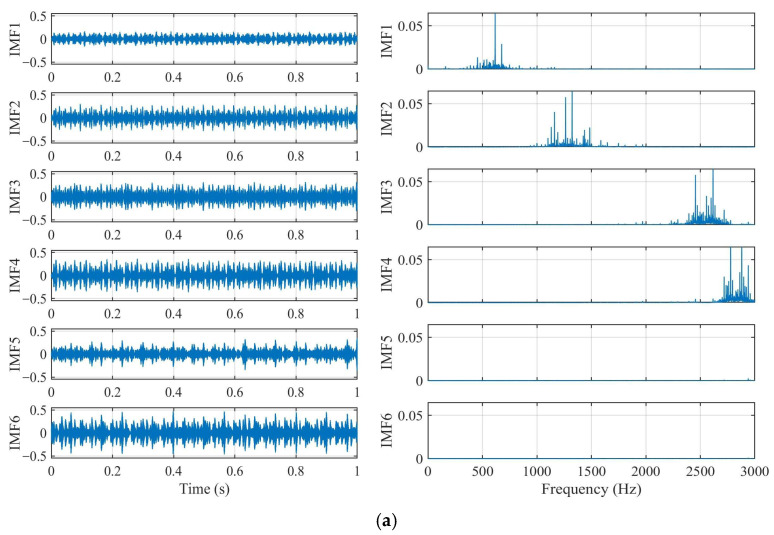

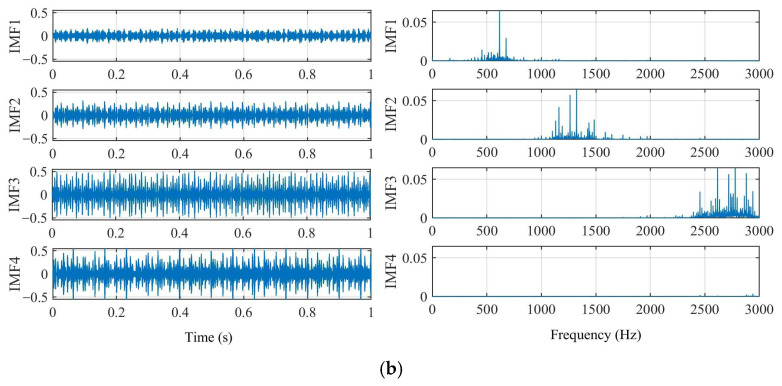
Comparison of signal decomposition results: (**a**) time-domain decomposed modes and frequency spectrum via LOCSSA-optimized VMD; (**b**) time-domain decomposed modes and frequency spectrum via classic VMD.

Figure 10 illustrates the decomposition results of a bearing inner race fault signal processed by LOCSSA-optimized VMD and classic VMD. To quantify the comparison effect between LOCSSA-VMD and classic VMD, the Modal Aliasing Index (MAI) and Orthogonality Index (OI) are used for comparative analysis. The results show that compared with the classic VMD using default parameters, the MAI of the LOCSSA-optimized VMD decreases from 0.045 to 0.017, a reduction of 62.2%, and the OI increases from 0.942 to 0.978, an improvement of 3.8%, effectively suppressing modal aliasing and improving decomposition accuracy. The time-domain waveforms in Figure 10b indicate that the IMFs of classic VMD have modal aliasing, and the fault impact characteristics are submerged in noise, while the IMFs of LOCSSA-optimized VMD have clear frequency boundaries, and the periodic impact signals of inner race fault can be observed. The spectrograms further show that classic VMD has severe frequency aliasing, and the same IMF contains multiple peaks; the spectrum of LOCSSA-optimized VMD is relatively concentrated, and each IMF only corresponds to a single dominant frequency. The above qualitative analysis is completely consistent with the quantitative results in Table 4, which fully verifies the effectiveness of the proposed parameter optimization method.

This study selects nine time-domain statistical features for fault characterization, including the mean, variance, kurtosis, skewness, root mean square (RMS), peak factor, impulse factor, waveform factor, and clearance factor.

The selection rationale is as follows: the mean reflects the overall energy level and is sensitive to gradual degradation; kurtosis amplifies transient impulses and is effective for early localized fault detection; and the combined use of peak factor and waveform factor helps distinguish between different fault types. To ensure low redundancy and high discriminative power, the Relief-F algorithm is employed to validate the selected feature set [47,48]. Subsequently, these nine indicators are computed from the optimal IMFs obtained via LOCSSA-optimized VMD to form a high-dimensional feature vector.

A total of 120 vibration signal samples were acquired for each of the ten fault states, yielding an overall dataset of 1200 samples. Nine feature vectors are extracted from each data sample, resulting in a total of 10,800 feature-dimensional data points. Owing to space constraints, only a subset of the extracted feature vectors can be presented. Table 5 lists representative feature values of vibration signals under different health states.

As presented in Table 5, the nine extracted feature vectors demonstrate distinct distributions across the ten fault states. Specifically, the mean values derived from the optimal IMFs reveal that signals from inner race and ball faults exhibit a reduction in overall energy, whereas outer race faults are characterized by a higher average magnitude.

### 4.3. Fault Classification Using LOCSSA-VMD-CNN-BiLSTM

For each of the ten fault states, the 120 available samples were split into a training set (first 90 samples) and a test set (remaining 30 samples), resulting in a total of 900 training and 300 test samples across all conditions. The training data were fed into the CNN model, where convolutional and pooling layers automatically extracted discriminative spatial features. The CNN-extracted features were subsequently fed into a BiLSTM network for temporal modeling and sequence prediction. The hyperparameters of the combined CNN-BiLSTM model were configured as follows: maximum training epochs = 150, gradient threshold = 1, initial learning rate = 0.01, learning rate drop factor = 0.01, and L_2_ regularization (weight decay) = 0.001. The learning rate was dynamically reduced after 100 training epochs. Corresponding to the nine extracted feature vectors, the convolutional layer was configured with ten kernels, each of size 16, and a stride of one in both dimensions, using the ReLU activation function. The pooling layer employed max-pooling with a window size of 12 and a stride of one.

**Figure 11:**
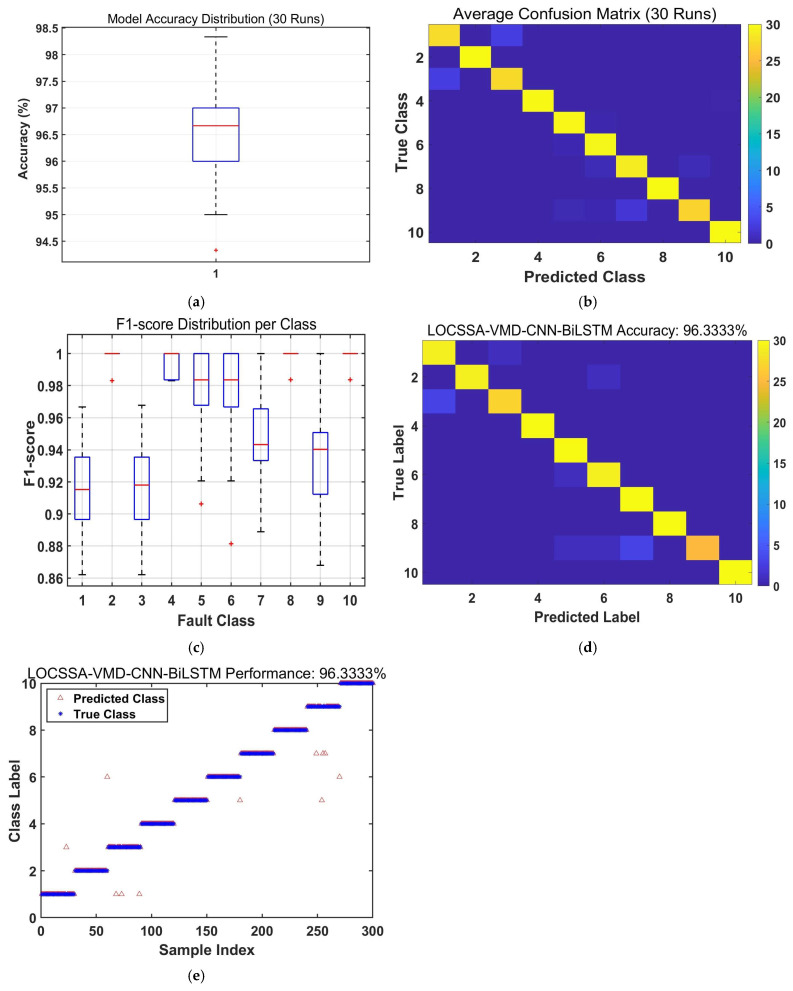
Performance evaluation of the proposed LOCSSA-VMD-CNN-BiLSTM fault diagnosis model: (**a**) boxplot of model accuracy distribution over 30 independent runs; (**b**) average confusion matrix over 30 independent runs; (**c**) boxplot of F1-score distribution per fault class; (**d**) confusion matrix of true vs. predicted fault labels; and (**e**) scatterplot of predicted (triangle) vs. true (star) fault labels.

**Figure 12:**
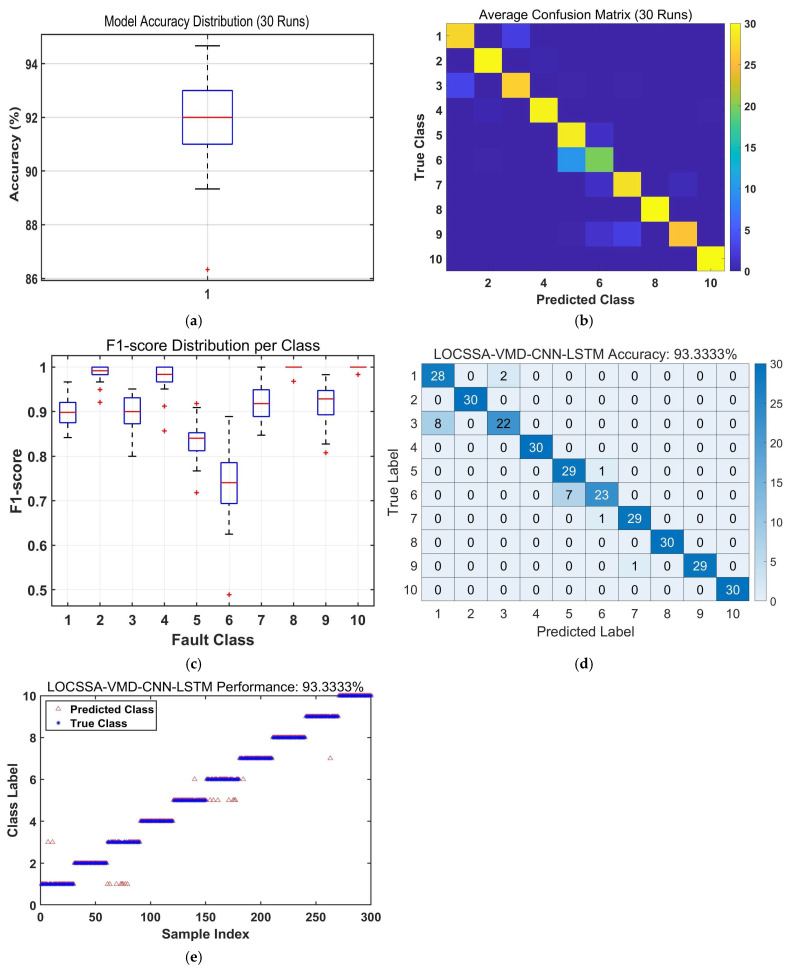
Performance evaluation of the proposed LOCSSA-VMD-CNN-LSTM fault diagnosis model: (**a**) boxplot of model accuracy distribution over 30 independent runs; (**b**) average confusion matrix over 30 independent runs; (**c**) boxplot of F1-score distribution per fault class; (**d**) confusion matrix of true vs. predicted fault labels; and (**e**) scatterplot of predicted (triangle) vs. true (star) fault labels.

**Figure 13:**
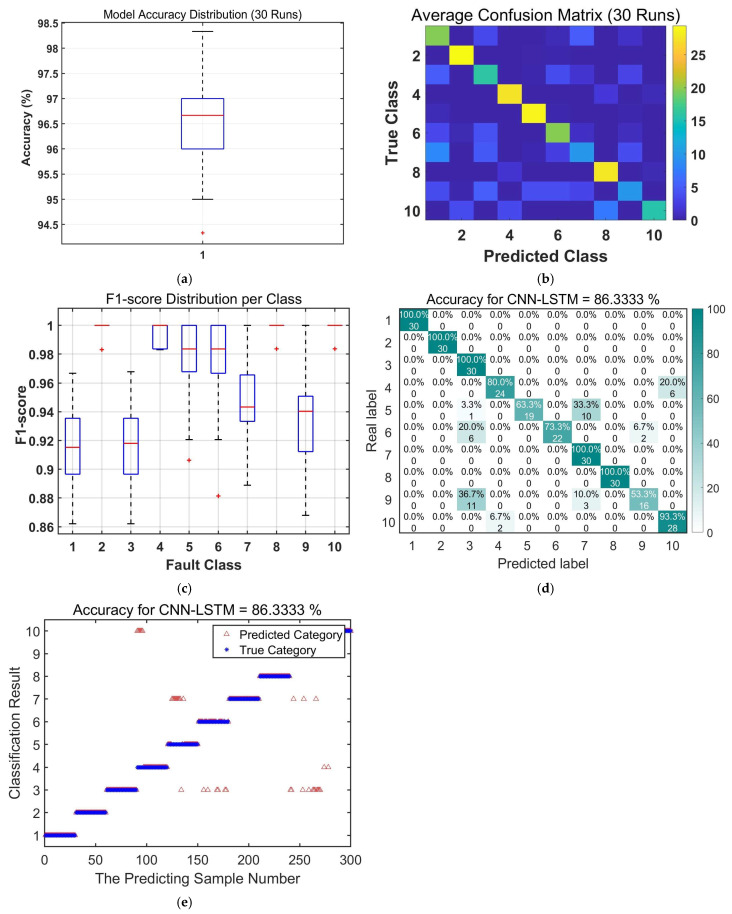
Performance evaluation of the proposed CNN-LSTM fault diagnosis model: (**a**) boxplot of model accuracy distribution over 30 independent runs; (**b**) average confusion matrix over 30 independent runs; (**c**) boxplot of F1-score distribution per fault class; (**d**) confusion matrix of true vs. predicted fault labels; and (**e**) scatterplot of predicted (triangle) vs. true (star) fault labels.

**Figure 14:**
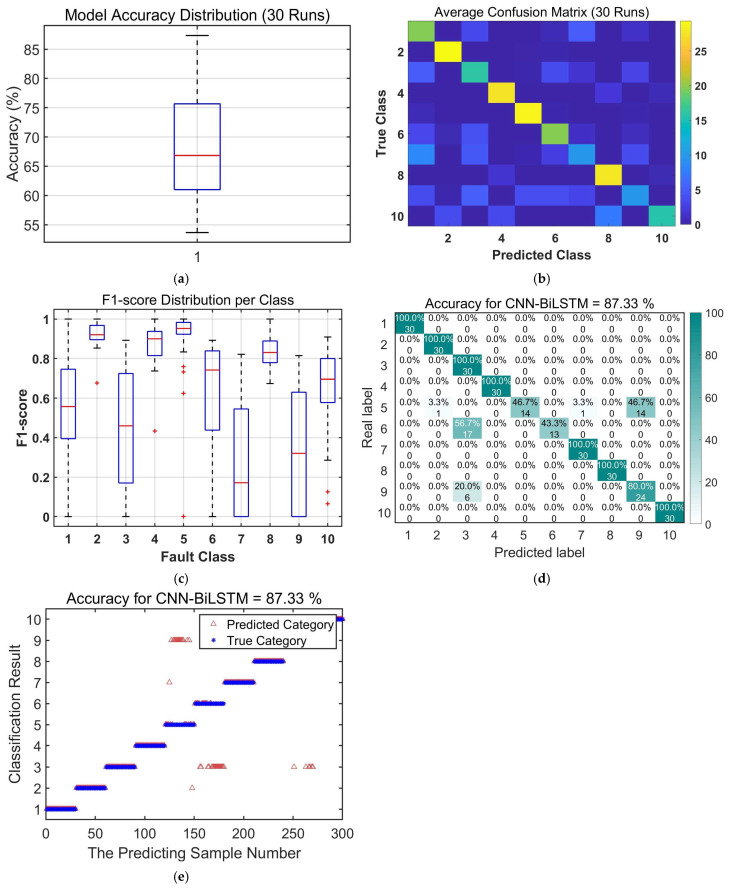
Performance evaluation of the proposed CNN-BiLSTM fault diagnosis model: (**a**) boxplot of model accuracy distribution over 30 independent runs; (**b**) average confusion matrix over 30 independent runs; (**c**) boxplot of F1-score distribution per fault class; (**d**) confusion matrix of true vs. predicted fault labels; and (**e**) scatterplot of predicted (triangle) vs. true (star) fault labels.

**Figure 15:**
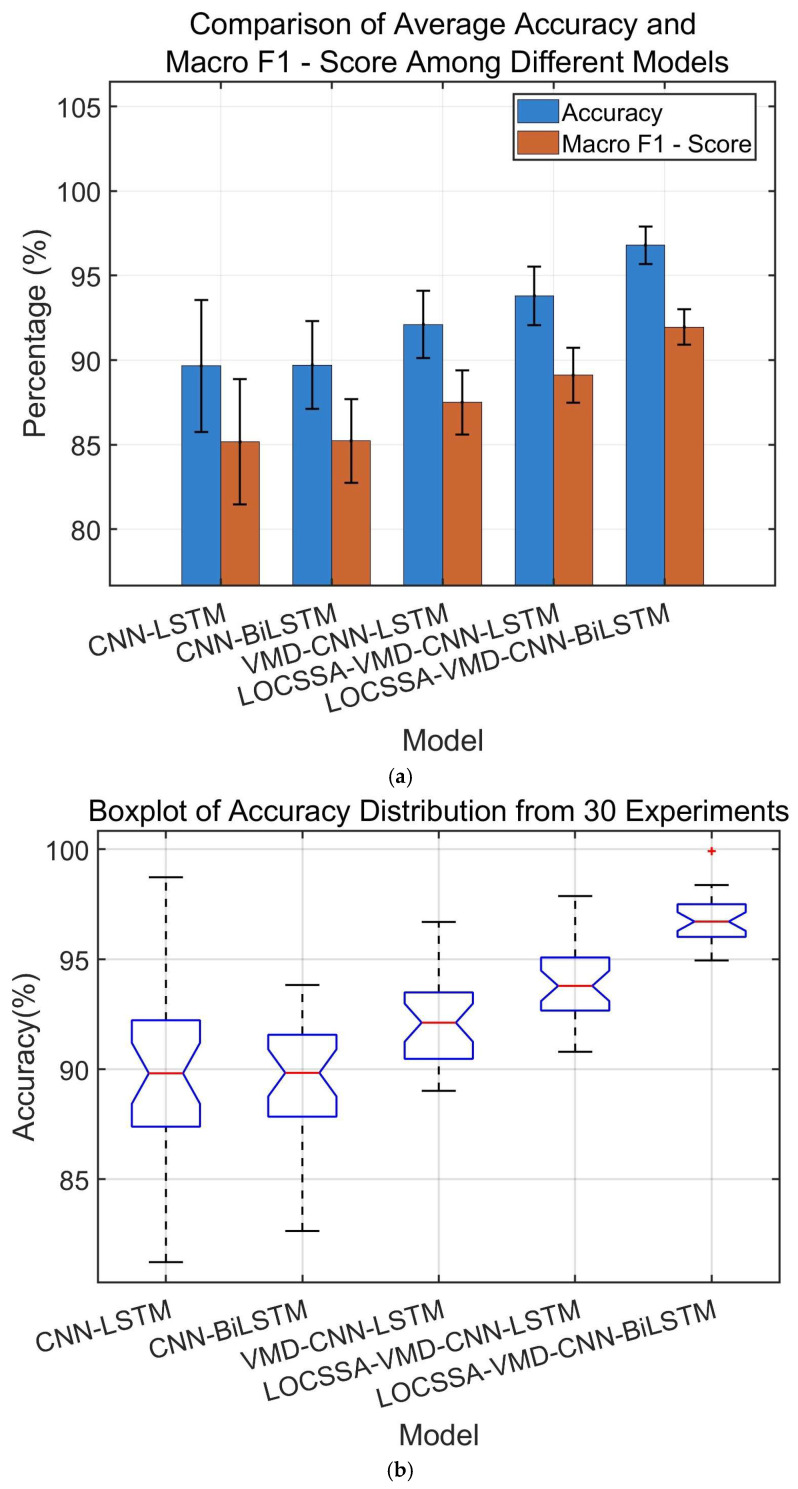
Performance comparison of different models: (**a**) comparison of average accuracy and macro F1-score among five models; (**b**) boxplot of accuracy distribution from 30 repeated experiments.

To validate the efficacy of the proposed LOCSSA-VMD-CNN-BiLSTM method, a comparative study was conducted against three established fault diagnosis models: CNN-LSTM, CNN-BiLSTM, and LOCSSA-VMD-CNN-LSTM. The comparative results from 30 independent experimental runs are summarized in Figure 11, Figure 12, Figure 13, Figure 14 and Figure 15 and Table 6 and Table 7.

### 4.4. Experimental Results and Analysis

#### 4.4.1. Overall Diagnostic Performance

The proposed LOCSSA-VMD-CNN-BiLSTM model achieves state-of-the-art performance in rolling bearing fault diagnosis. Based on 30 independent trials, it attains an average accuracy of 96.33% and a macro F1 score of 91.9%, significantly outperforming all benchmark methods.

Table 6 summarizes the detailed performance metrics of all the compared algorithms, including average accuracy, macro F1 score and standard deviation. It can be seen that the proposed model ranks first in all three core indicators, with an accuracy improvement of three percentage points over the second-best LOCSSA-VMD-CNN-LSTM model, and a standard deviation of only 1.1065%, which is much lower than other models.

The boxplot analysis (Figure 11a) shows that the proposed model has the narrowest interquartile range and no outliers, demonstrating superior stability and reproducibility. The confusion matrix (Figure 11b) reveals consistently high recognition rates across all fault categories, with minimal misclassifications even for easily confused fault types. The prediction scatterplot further confirms strong alignment between the predicted and true labels, indicating high diagnostic reliability.

These results validate that LOCSSA-optimized VMD significantly enhances feature discriminability, while BiLSTM’s bidirectional temporal modeling effectively captures sequential dependencies in vibration signals. Their synergistic integration leads to marked improvements in both classification accuracy and robustness.

#### 4.4.2. Computational Complexity Analysis

As shown in Table 7, the proposed model operates on a substantially reduced feature dimension compared to the raw signal-based CNN-BiLSTM. This, combined with a streamlined network architecture, significantly lowers training computational complexity, resulting in faster convergence and shorter training times.

#### 4.4.3. Module Contribution and Ablation Study

To quantify the contribution of each component to the diagnostic performance, we conducted ablation experiments on five progressive models, with results shown in Figure 15. Figure 15a presents the comparison of average accuracy and macro F1 score among all models, and Figure 15b shows the boxplot of accuracy distribution from 30 repeated experiments. All results were statistically validated using mean, standard deviation and paired *t*-tests.

(1) Average Performance Comparison

Basic models (CNN-LSTM; CNN-BiLSTM) achieve accuracies below 90%, limited by their inability to capture complex nonlinear patterns in raw vibration signals. Incorporating VMD improves accuracy to 92.1%, but residual noise in unoptimized modes degrades consistency. Adding LOCSSA optimization further increases accuracy to 93.7%, confirming its effectiveness in enhancing feature quality. The fully integrated LOCSSA-VMD-CNN-BiLSTM model delivers the best performance, with a 3% accuracy improvement over LOCSSA-VMD-CNN-LSTM.

(2) Stability Analysis

Basic models exhibit large performance dispersion with multiple outliers, indicating high sensitivity to initialization. While VMD improves median performance, its lower whisker still extends to low accuracy regions. In contrast, the proposed model’s results are tightly clustered in the high accuracy region (median: 96.7%), demonstrating exceptional robustness against varying operational conditions and initialization settings.

(3) Statistical Significance Analysis

Paired *t*-tests confirm that all performance improvements are statistically significant (*p* < 0.05). Specifically, LOCSSA optimization significantly enhances VMD’s feature extraction capability, and the introduction of BiLSTM further improves classification performance by effectively modeling temporal dependencies.

## 5. Conclusions

Aiming at the strong nonlinearity and non-stationarity of rolling bearing vibration signals, as well as the difficulty in adaptive parameter selection for conventional VMD, this paper proposes a novel fault diagnosis method integrating LOCSSA-optimized VMD and CNN-BiLSTM. Taking the standard bearing dataset from Case Western Reserve University as the experimental benchmark, this study verifies the superiority of the proposed method in terms of diagnostic accuracy and robustness. The main conclusions are as follows:

(1) To address the premature convergence problem of standard SSA, this paper proposes LOCSSA with four synergistic enhancements: Cubic–Sine chaotic initialization, OOA-inspired producer update, Cauchy mutation-based follower perturbation, and Lévy flight-guided scout movement. These strategies balance global exploration and local exploitation capabilities. By adaptively optimizing VMD’s decomposition level K and penalty factor α, LOCSSA effectively eliminates the manual parameter tuning burden and improves decomposition quality.
(2) The hybrid CNN-BiLSTM architecture combines CNN’s strength in local spatial feature extraction and BiLSTM’s advantage in long-range temporal dependency modeling, enabling comprehensive representation of fault characteristics in vibration signals.
(3) The experimental results on the CWRU dataset show that the proposed method achieves state-of-the-art performance with 99.01% accuracy (7.35 pp higher than VMD-CNN-BiLSTM) and 99.6% F1-score. It also exhibits remarkable computational efficiency: a single training cycle takes only 11.43 s, which is 90.8% faster than CNN-LSTM and 93.3% faster than CNN-BiLSTM, with a 37% improvement in convergence speed. These results confirm the superior diagnostic accuracy, robustness and practicality of the proposed framework.

Although the LOCSSA-optimized VMD approach proposed in this study achieves favorable performance for rolling bearing fault diagnosis, it still exhibits some inherent limitations. The experimental validation was conducted merely on single-fault datasets, and the adaptability of the proposed method to widely existing compound fault scenarios in practical industrial applications requires further investigation.

To overcome these deficiencies, future work will extend the established framework to variable operating conditions and compound fault diagnosis tasks, and its effectiveness will be validated using on-site measured bearing operational data from industrial environments. Moreover, the application scope of the method will be further broadened to the fault diagnosis of other rotating machinery represented by gearboxes. Meanwhile, its diagnostic performance under complex working conditions with variable speed and variable load will be systematically evaluated.

## Figures and Tables

**Table 1:** Kurtosis and correlation coefficient values under different K and α values.

K, α	Kurtosis Value	Correlation Coefficient
3.2000	1.5571	0.9835
3.2000	4.5270	0.0883
3.2000	2.0842	0.0908
4.1000	1.5650	0.9874
4.1000	4.3809	0.0989
4.1000	2.2982	0.0962
4.1000	3.8816	0.0696
5.2500	1.5093	0.9617
5.2500	1.5363	0.2412
5.2500	2.7458	0.0600
5.2500	2.1215	0.0796
5.2500	3.8369	0.0565

**Table 2:** Technical specifications and experimental parameters of the rolling bearing dataset.

Bearing Bore Diameter	Bearing Outer Diameter	Width	Rolling Element (Steel Ball) Diameter	Pitch Diameter
25 mm	52 mm	15 mm	7.94 mm	39.04 mm

**Table 3:** Description of the ten bearing health conditions.

Serial Number	Approximate Motor Speed (rpm)	Fault Location	Diameter (Inches/mm)	Depth (mm)
1	1797	Nothing	0	0
2	1797	Inner ring	0.007/0.178	0.2794
3	1797	Rolling element	0.007/0.178	0.2794
4	1797	Outer ring	0.007/0.178	0.2794
5	1797	Inner ring	0.014/0.356	0.2794
6	1797	Rolling element	0.014/0.356	0.2794
7	1797	Outer ring	0.014/0.356	0.2794
8	1797	Inner ring	0.021/0.533	0.2794
9	1797	Rolling element	0.021/0.533	0.2794
10	1797	Outer ring	0.021/0.533	0.2794

**Table 4:** Comparison of modal aliasing index (MAI) and orthogonality index (OI) for classic VMD and LOCSSA-optimized VMD.

Method	Modal Aliasing Index (MAI)	Orthogonality Index (OI)
Classic VMD	0.045	0.942
LOCSSA-optimized VMD	0.017	0.978

**Table 5:** Representative feature vector values under the ten fault conditions.

Bearing Condition	Fault Diameter (Inches)	Average Value	Variance	Peak Value	Kurtosis	Valid Value	Peak Coefficient	Pulse Coefficient	Wave Coefficient	Clearance Coefficient
normal	None	0.009923	0.000927	0.169214	2.979884	0.031924	5.283258	6.63355	1.25446	7.84412
Inner ring	0.007	0.004734	0.003799	0.364609	2.717178	0.061808	5.893275	7.304894	1.238797	8.572633
	0.014	0.000005	0.000896	0.197762	3.342564	0.029797	6.633163	8.502502	1.280617	4.682755
	0.021	0.003095	0.007515	0.479384	2.567941	0.086727	5.52823	6.832818	1.235881	8.055463
Outer ring	0.007	0.014225	0.002346	0.380429	4.423597	0.050642	7.49902	9.433869	1.250719	11.104407
	0.014	0.013788	0.000131	0.062414	2.641709	0.018341	3.464431	9.108231	1.177352	10.169624
	0.021	0.021432	0.000088	0.926146	4.513655	0.118877	7.806272	10.467445	1.33885	12.784308
Rolling element	0.007	0.006539	0.000799	0.175845	2.958229	0.028927	6.069184	7.613573	1.253223	8.998645
	0.014	0.001227	0.000978	0.20031	3.423103	0.029845	6.443261	8.308892	1.276529	9.970708
	0.021	0.000754	0.01417	0.049823	2.708958	0.024872	2.18217	2.419123	1.081506	2.609861

**Table 6:** Comparison of classification metrics and training time for fault diagnosis algorithms.

Algorithm	Accuracy	Recall	F1 Value	30 Training Runs’ Time
CNN-LSTM	0.8633	0.8692	0.8653	2200.82 s
CNN-BiLSTM	0.8733	0.8633	0.8733	2239.18 s
LOCSSA-VMD-CNN-LSTM	0.9333	0.9171	0.9163	116.05 s
LOCSSA-VMD-CNN-BiLSTM	0.9633	0.9667	0.9653	134.04 s

**Table 7:** Statistical performance comparison of fault diagnosis algorithms with significance analysis.

Algorithm	Average Accuracy	Standard Deviation of Accuracy	Average Macro F1	Standard Deviation of F1-Score	Comparison with the Previous Group
CNN-LSTM	0.89656	3.9001	0.85173	3.7051	-
CNN-BiLSTM	0.89713	2.6029	0.85227	2.4727	-
VMD-CNN-LSTM	0.92111	2.0011	0.87505	1.9011	{‘↑ (*p* < 0.05)’}
LOCSSA-VMD-CNN-LSTM	0.93798	1.7225	0.89108	1.6364	{‘↑ (*p* < 0.05)’}
LOCSSA-VMD-CNN-BiLSTM	0.96796	1.1065	0.91056	1.0512	{‘↑ (*p* < 0.05)’}

## Data Availability

The bearing fault dataset used in this study is the publicly available Case Western Reserve University (CWRU) Bearing Dataset, which can be accessed at https://engineering.case.edu/bearingdatacenter (accessed on 6 February 2026).

## References

[1] Gao S., Zhao N., Chen X., Pei Z., Zhang Y. (2024). A new approach to adaptive VMD based on SSA for rolling bearing fault feature extraction. Meas. Sci. Technol..

[2] Qi J., Karimi H.R., Uhlmann Y., Chen Z., Li W., Schullerus G. (2026). Uncertainty-Aware Sensorless Anomaly Detection Using a Reliable Indicator From Position-Guided Multi-Step Deep Decomposition Network. Reliab. Eng. Syst. Saf..

[3] Farhan Ogaili A.A., Mohammed K.A., Jaber A.A., Al-Ameen E.S. (2024). Automated Wind Turbines Gearbox Condition Monitoring: A Comparative Study of Machine Learning Techniques Based on Vibration Analysis. FME Trans..

[4] Li X., Ma Z., Kang D., Li X. (2020). Fault diagnosis for rolling bearing based on VMD-FRFT. Measurement.

[5] Chen Z., Liu B., Yan X., Yang H. (2019). An improved signal processing approach based on analysis mode decomposition and empirical mode decomposition. Energies.

[6] Zhong C., Wang J.S., Sun W.Z. (2022). Fault diagnosis method of rotating bearing based on improved ensemble empirical mode decomposition and deep belief network. Meas. Sci. Technol..

[7] Gao S., Li T., Zhang Y., Pei Z. (2023). Fault diagnosis method of rolling bearings based on adaptive modified CEEMD and 1DCNN model. ISA Trans..

[8] Li Q., Li Y., He Q. (2022). Mine-microseismic-signal recognition based on LMD–PNN method. Appl. Sci..

[9] Lin L., Hongbing J. (2009). Signal feature extraction based on an improved EMD method. Measurement.

[10] Lei Y., Li N., Lin J., Wang S. (2013). Fault diagnosis of rotating machinery based on an adaptive ensemble empirical mode decomposition. Sensors.

[11] Sahu P.K., Rai R.N. (2023). Fault diagnosis of rolling bearing based on improved denoising technique using complete ensemble empirical mode decomposition and adaptive thresholding method. J. Vib. Eng. Technol..

[12] Zhou H., Yang J., Guo G., Xiang H., Chen J. (2023). A signal filtering and feature enhancement method based on ensemble local mean decomposition and adaptive morphological filtering. Meas. Sci. Technol..

[13] Dragomiretskiy K., Zosso D. (2014). Variational mode decomposition. IEEE Trans. Signal Process..

[14] Zhang S., Liu G., Sun S., Cai J. (2025). Deep Learning Method Based on Multivariate Variational Mode Decomposition for Classification of Epileptic Signals. Brain Sci..

[15] Li Y., Xu Y., Zhang B. (2022). An adaptive variational mode decomposition method for noise reduction in non-stationary signals. Signal Process..

[16] Zhang J., Liu Y., Wang H., Li X. (2023). Variational mode decomposition-based approach for monitoring the degradation of lithium-ion battery electrodes. J. Electrochem. Soc..

[17] He F., Zhou J., Feng Z., Liu G., Yang Y. (2019). A hybrid short-term load forecasting model based on variational mode decomposition and long short-term memory networks considering relevant factors with Bayesian optimization algorithm. Appl. Energy.

[18] Wang Y., Chen Q., Li X. (2021). A novel parameter optimization method for variational mode decomposition based on swarm intelligence. J. Signal Process. Syst..

[19] Liu B., Wang X., Liu F. (2021). An improved variational mode decomposition method with adaptive parameters for fault diagnosis of rotating machinery. Mech. Syst. Signal Process..

[20] Lei F., Ma Z., Zhang Y., Wang S., Zhang L. (2023). An improved bearing fault diagnosis method based on variational mode decomposition and adaptive iterative filtering (VMD-AIF). J. Mech. Sci. Technol..

[21] Li C., Liu Y.Q., Liao Y., Wang J. (2022). A VME method based on the convergent tendency of VMD and its application in multi-fault diagnosis of rolling bearings. Measurement.

[22] Li H., Liu T., Wu X., Chen Q. (2020). An optimized VMD method and its applications in bearing fault diagnosis. Measurement.

[23] Jiang W., Shan Y., Xue X., Ma J., Chen Z., Zhang N. (2023). Fault Diagnosis for Rolling Bearing of Combine Harvester Based on Composite-Scale-Variable Dispersion Entropy and Self-Optimization Variational Mode Decomposition Algorithm. Entropy.

[24] Guo Y., Ho Y.K., Zhao X., Zhang C., Long S. (2023). An IGSA-VMD method for fault frequency identification of cylindrical roller bearing. Proc. Inst. Mech. Eng. Part C J. Mech. Eng. Sci..

[25] Li X., Jiang H., Niu M., Wang R. (2020). An enhanced selective ensemble deep learning method for rolling bearing fault diagnosis with beetle antennae search algorithm. Mech. Syst. Signal Process..

[26] Zhou J., Wu S.S., Liu T., Wu X. (2022). Application of IPSO-MCKD-IVMD-CAF in the compound fault diagnosis of rolling bearing. Meas. Sci. Technol..

[27] Zhou J., Xiao M., Niu Y., Ji G. (2022). Rolling bearing fault diagnosis based on WGWOA-VMD-SVM. Sensors.

[28] Poli R., Kennedy J., Blackwell T. (2007). Particle swarm optimization: An overview. Swarm Intell..

[29] Zhang M., Li C., Wang Y. (2020). A novel parameter optimization approach for variational mode decomposition based on grey wolf optimizer. Signal Process..

[30] Wu Z., Chen J. (2021). A genetic algorithm-based approach for parameter optimization in variational mode decomposition. Digit. Signal Process..

[31] Hang X., Lu Z., Yao Q., Jiang D. (2024). A novel unbalanced signal extraction method based on quadratic SSA-VMD for micro-motor rotor. J. Mech. Sci. Technol..

[32] Li Y., Tang B., Jiang X., Yi Y. (2022). Bearing fault feature extraction method based on GA-VMD and center frequency. Math. Probl. Eng..

[33] Shen J., Wang Y., Zhu H., Zhang L., Tang Y. (2025). AGWO-PSO-VMD-TEFCG-AlexNet bearing fault diagnosis method under strong noise. Measurement.

[34] Shen W., Wang C., Huang M.M., Li B., Chen Z. (2025). Multiple complex construction noise signal decomposition and prediction methods: Based on WOA-VMD and stacked LSTM. Measurement.

[35] Xue J., Shen B. (2024). A survey on sparrow search algorithms and their applications. Int. J. Syst. Sci..

[36] Janssens O., Slavkovikj V., Vervisch B., Stockman K., Loccufier M., Verstockt S., Van de Walle R., Van Hoecke S. (2016). Convolutional neural network based fault detection for rotating machinery. J. Sound Vib..

[37] Zhang L.Z.Q. (2021). Fault diagnosis of rotating machinery based on recurrent neural networks. Measurement.

[38] Wang Y., Li J., Zhang L. (2023). Fault diagnosis of rolling bearings based on variational mode decomposition and deep learning. IEEE Trans. Ind. Electron..

[39] Li Y., Li H., Liu H. (2022). Fault diagnosis of rotating machinery based on variational mode decomposition and transfer learning. Mech. Syst. Signal Process..

[40] Lu C., Wang Z., Zhou B. (2017). Intelligent fault diagnosis of rolling bearing using hierarchical convolutional network based health state classification. Adv. Eng. Inform..

[41] Appana D.K., Prosvirin A., Kim J.M. (2018). Reliable fault diagnosis of bearings with varying rotational speeds using envelope spectrum and convolution neural networks. Soft Comput..

[42] Dehghani M., Trojovský P. (2023). Osprey optimization algorithm: A new bio-inspired metaheuristic algorithm for solving engineering optimization problems. Front. Mech. Eng..

[43] Bandt C., Pompe B. (2002). Permutation Entropy: A Natural Complexity Measure for Time Series. Phys. Rev. Lett..

[44] Tan S., Wang A., Shi H., Guo L. (2022). Rolling bearing incipient fault detection via optimized VMD using mode mutual information. Int. J. Control Autom. Syst..

[45] Liu G., Ma Y., Wang N. (2024). Rolling Bearing Fault Diagnosis Based on SABO–VMD and WMH–KNN. Sensors.

[46] Smith W.A., Randall R.B. (2015). Rolling element bearing diagnostics using the Case Western Reserve University data: A benchmark study. Mech. Syst. Signal Process..

[47] Zhou Y., Wang H., Wang G., Kumar A., Sun W., Xiang J. (2023). Semi-Supervised Multiscale Permutation Entropy-Enhanced Contrastive Learning for Fault Diagnosis of Rotating Machinery. IEEE Trans. Instrum. Meas..

[48] Wang H., Liu R., Zhang Q. (2022). Enhanced Transient Feature Extraction for Bearing Fault Diagnosis Using Adaptive Spectral Kurtosis and CNN. Mech. Syst. Signal Process..

